# Ultraviolet absorption of contaminants in water

**DOI:** 10.1038/s41598-021-83322-w

**Published:** 2021-02-11

**Authors:** Martin Spangenberg, James I. Bryant, Sam J. Gibson, Philip J. Mousley, Yorck Ramachers, Gavin R. Bell

**Affiliations:** 1 Department of Physics, University of Warwick, Coventry, CV4 7AL UK; 2 Present Address: Centre for Engineering Photonics, Cranfield University, Cranfield, MK43 0AL UK; 3 Present Address: Diamond Light Source, Harwell, OX11 0DE UK

**Keywords:** Environmental sciences, Techniques and instrumentation

## Abstract

Contaminants in water were studied using ultraviolet absorption with light emitting diode and deuterium lamp sources, and a thresholding detector. The absorption spectra of potassium hydrogen pthalate, clothianidin, tryptophan, thiamethoxam, uric acid and metaldehyde were obtained in the range 200–360 nm. Only metaldehyde was not suitable for detection in this range. For the other contaminants, and mixtures of pairs of compounds, the transmitted signal could be approximately described with a simple spectral model of the source–absorption–detector system. Combined measurements at two wavelengths could allow relative concentrations in certain mixtures to be determined, and real-time absorption measurements were demonstrated in a flume.

## Introduction

The contamination of water by human activity is a major environmental and health concern^1^. Contaminants include pesticides^2,3^, heavy metals^4^ and sewage^5^ while many other emerging pollutants are of concern^6,7^, such as pharmaceuticals and personal care products^8^. Significant worry has arisen over the adverse effects of neonicotinoid pesticides such as clothianidin and thiamethoxam on bees and other pollinators, and these are included in EU-wide watch-lists for monitoring in surface water^9^. Detection methods for pollutants in water include sophisticated analytical approaches such as liquid chromatography^10^, electrode potentiometry^11,12^, mass spectrometry^13^, UV–Vis spectrometry^14^ and Raman scattering^15^. However, laboratory data are not necessarily needed to provide valuable inputs to water quality monitoring and control schemes^16^. Simpler methods can also be very useful, particularly where there are practical or economic constraints on sampling, transport to a laboratory setting, etc. There is increasing interest in distributed and continuous water quality monitoring using remote sensors for parameters such as resistivity, temperature, pH, total suspended solids (TSS), absorbance at 254 nm (UV-254) and total organic content (TOC)^17–19^. Such devices can provide rapidly sampled information on the behaviour of contaminants and biochemical processes in habitats, distribution networks, catchment areas and industrial settings^20–23^. The data can be used as early-warning alerts^24^, on water network or process management “dashboards” or to manage water systems via fuzzy logic^16^ or machine learning.

Optical methods are widely used. UV–Vis spectrometry uses a dispersing spectrometer to measure absorbance as a function of wavelength^14,25^. Fluorescence and Raman scattering are also used, again with a dispersing spectrometer^15,26^. While field-deployable spectrometers are available, they are relatively expensive and so simpler single-wavelength absorbance devices could be attractive for widespread sensing networks. The standard UV-254 parameter relates to absorbance at 254 nm only but water is reasonably transparent over a wide range of wavelengths into the visible. Pure water has a scattering-independent absorption coefficient below 0.1 m $^{-1}$ in the range 250 nm to 350 nm, with a minimum below 10 $^{-3}$ m $^{-1}$ at the upper end of this range^27^. Absorption and scattering are strongly influenced by dissolved substances, suspended particulates, bubbles and other impurities^28,29^, making inherent optical properties difficult to extract reliably. In the low-absorption regime such effects can be exploited in the optical detection of water contamination. The complexity of natural water means that such measurements should ideally account for both scattering and absorption at different wavelengths. However, as inputs for fuzzy logic or similar real-time analysis of water systems, “naive” absorbance measurements at multiple wavelengths could be extremely useful. The advent of efficient and increasingly affordable light emitting diodes (LEDs) spanning the range 250 nm to 350 nm suggests that multi-wavelength UV measurements could be made without resorting to dispersing spectrometry. The choice of light detector then becomes important: good responsivity in the chosen wavelength ranges is important but too broad a detection band, particularly one extending into the visible range, could increase background signal and hence limit sensitivity.

Here we present UV absorption data for several water contaminants of practical interest. We combine absorption spectra obtained using a conventional UV–Vis spectrometer and selected-wavelength transmittance measurements using LEDs and a wavelength-thresholding UV detector whose photocathode operates in inert gas^30^. It is shown that the spectral discrimination of even such a simple apparatus could be sufficient to usefully distinguish certain contaminant substances.

## Results

**Figure 1:**
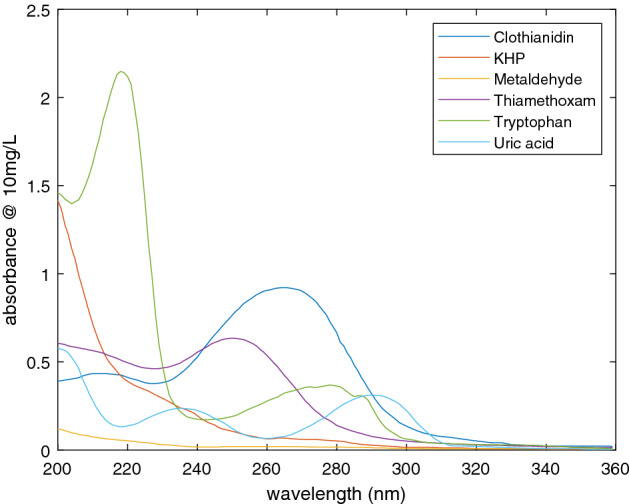
Absorption spectra for metaldehyde, clothianidin, tryptophan, thiamethoxam, potassium hydrogen pthalate (KHP) and uric acid, at 10 mg/L for 10 mm optical path.

UV absorption spectra were measured for a range of contaminants in water (concentration 10 mg/L, optical path 10 mm in silica glass cuvettes). These are shown in Fig. 1 for six substances relevant to water quality. All are transparent in the soft UV and visible range. Clothianidin and thiamethoxam are neonicotinoid pesticides. Each has a pair of broad absorption features below 280 nm. Tryptophan is an amino acid whose UV fluorescence is similar to that of organic matter such as sewage and farm slurry^31^. This shows a broad peak around 280 nm (absorption here is also associated with fluorescence at 340 nm) and a sharper peak at 220 nm. Potassium hydrogen pthalate (KHP) is used as a calibration standard for TOC measurements, and shows a steady increase of UV absorption below 250 nm. Metaldehyde, a pesticide for slugs and snails, absorbs rather weakly across the UV range studied and we found that it was not possible to reliably detect this compound with UV LEDs of wavelength 250 nm and above. Uric acid is present in untreated sewage but is not normally found in unpolluted waters^32^. It shows broad peaks at around 235 and 290 nm, and a peak or edge at 200 nm.

**Figure 2:**
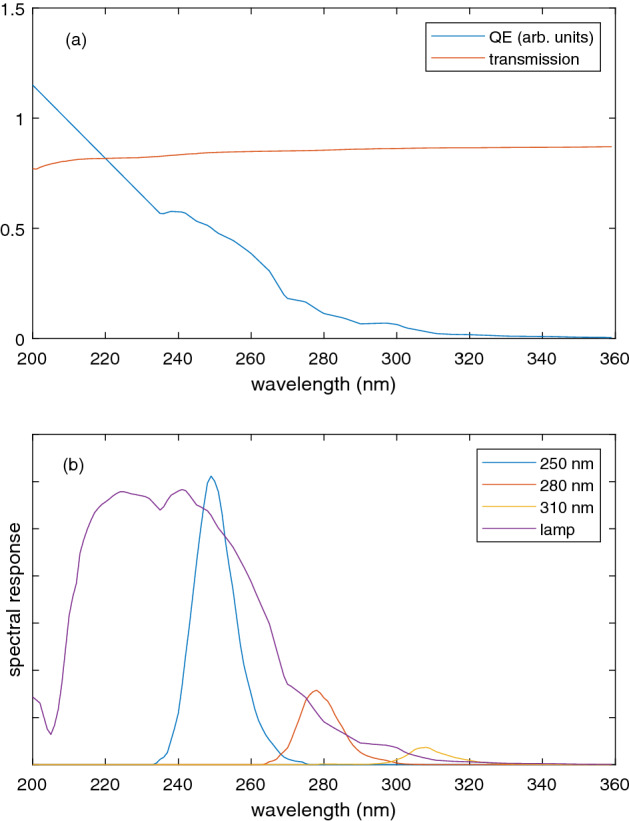
Spectral characteristics of the UV transmission apparatus. Panel (**a**) shows the transmittance and detector quantum efficiency, while (**b**) shows the spectral response of the light source–cuvette–detector system for three LEDs and a deuterium lamp.

Total transmittance measurements were made as a function of contaminant concentration using a simple light source and wavelength-thresholding detector. The same cuvettes were used as for UV–Vis measurements. The detector is based on a gaseous electron multiplier (GEM) similar to those developed for use as large-area detectors in particle physics experiments^33–35^. The responsivity of the UV detector depends on its quantum efficiency (QE) and electron multiplication gain. The relative QE for a typical GEM detector was measured and is shown in Fig. 2a, together with the estimated transmittance of the water and cuvette walls. Note that the precise QE curve varies slightly from one GEM to another, but the main shared features are (1) zero QE above a threshold corresponding to the work function and (2) steadily increasing QE below threshold and (3) similarity to a reported quantum yield curve for Cu^36^. The gain varies slightly from experiment to experiment but is a constant across the spectrum. The detector automatically filters out visible light and soft UV light. Note that we have linearly extrapolated the QE curve below the measurement limit of 235 nm (this only affects measurements made with a deuterium lamp, being below the LED wavelength range).

Figure 2b shows the combined spectral characteristics for the light source–cuvette–detector system, modelled by multiplying the spectrum of the light source by the transmittance and QE. Manufacturer data were used to define the light source spectra for a deuterium lamp and 3 LEDs with centre wavelengths 250, 280 and 310 nm. Because the QE is low near the threshold of the GEM detector, the spectral response of the 310 nm LED is weakest. It grows progressively larger for the 280 and 250 nm LEDs, with little overlap, and is strongest but broadest for the deuterium lamp. This suggests that even simple transmittance measurements at two wavelengths could provide some spectral information about substances with absorption peaks or edges in the 200–300 nm range.

**Figure 3:**
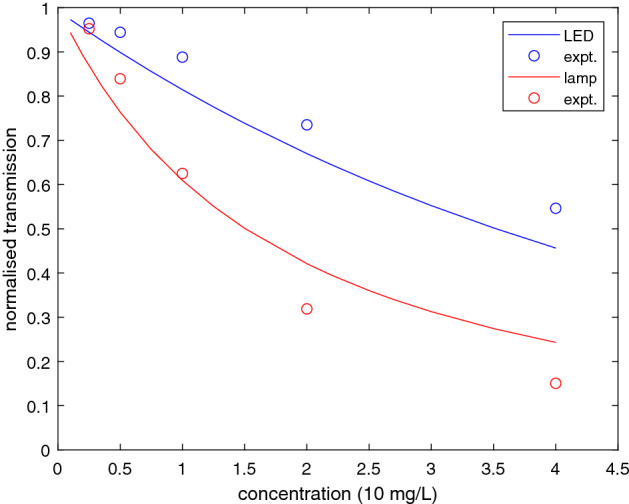
Total transmittance for KHP in water at different concentrations using a 250 nm LED and a deuterium lamp. Solid lines are from the model. Experimental error bars in both transmission and concentration are within the size of the symbols.

Figure 3 shows total normalised transmitted intensity for KHP at different concentrations using two light sources. It is immediately clear that the transmitted intensity decreases with increasing concentration much more quickly for illumination by the deuterium lamp than for the 250 nm LED. This is explained by the different overlap between the spectral response function (Fig. 2) and the absorption spectrum (Fig. 1). The short wavelength intensity of the lamp includes much of the increasing absorbance of KHP below 240 nm, whereas the 250 nm LED only overlaps with the weaker absorbance shoulder above 240 nm. The simple, parameter-free model describing this effect is detailed in the next section.

**Figure 4:**
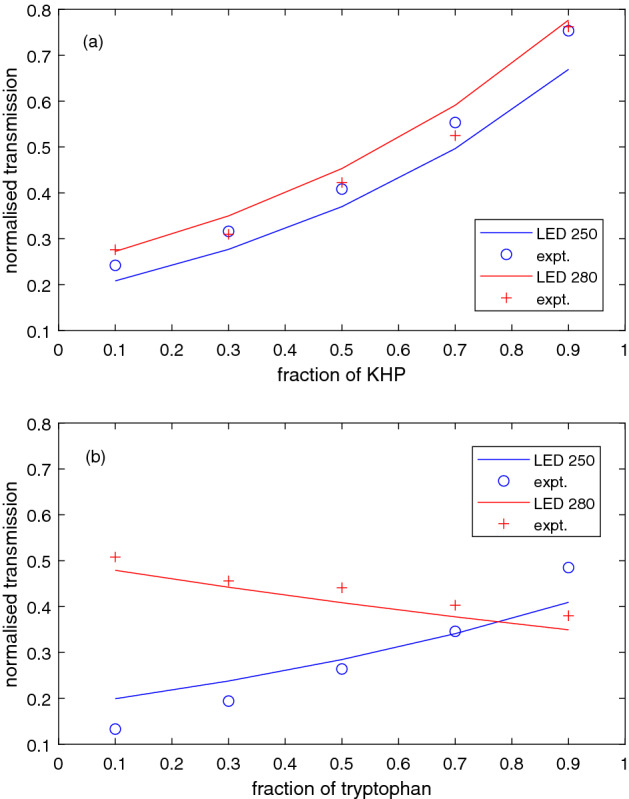
Transmittance data (symbols) and model (lines) for two mixtures of contaminants in water, with total concentration 10 mg/L and using 250 nm (blue) and 280 nm (red) LEDs. The mixtures are (**a**) KHP and clothianidin, and (**b**) tryptophan and thiamethoxam.

In order to examine the effects on total transmittance of mixtures of contaminants, two pairs of contaminants were mixed in different ratios to a total concentration of to 10 mg/l in water. These were KHP–clothainidin and tryptophan–thiamethoxam, and transmittance data are shown in Fig. 4a,b respectively. Both 250 and 280 nm LEDs were used. In the case of KHP–clothainidin, as the KHP fraction increases the transmittance at both wavelengths increases. This is due to the low absorbance of the KHP across the 250–280 nm range compared to clothianidin, which has a strong peak around 265 nm. The normalised response of the 250 nm and 280 nm LEDs is very similar since they roughly straddle this broad absorption peak. In contrast, the transmittance of the tryptophan–thiamethoxam mixtures behaves differently at the two wavelengths. As the fraction of tryptophan increases, the 250 nm transmittance increases since the absorption of the broad thiamethoxam peak around 250 nm decreases. Conversely, the 280 nm transmittance drops due to increasing absorption by the broad tryptophan peak around 280 nm.

**Figure 5:**
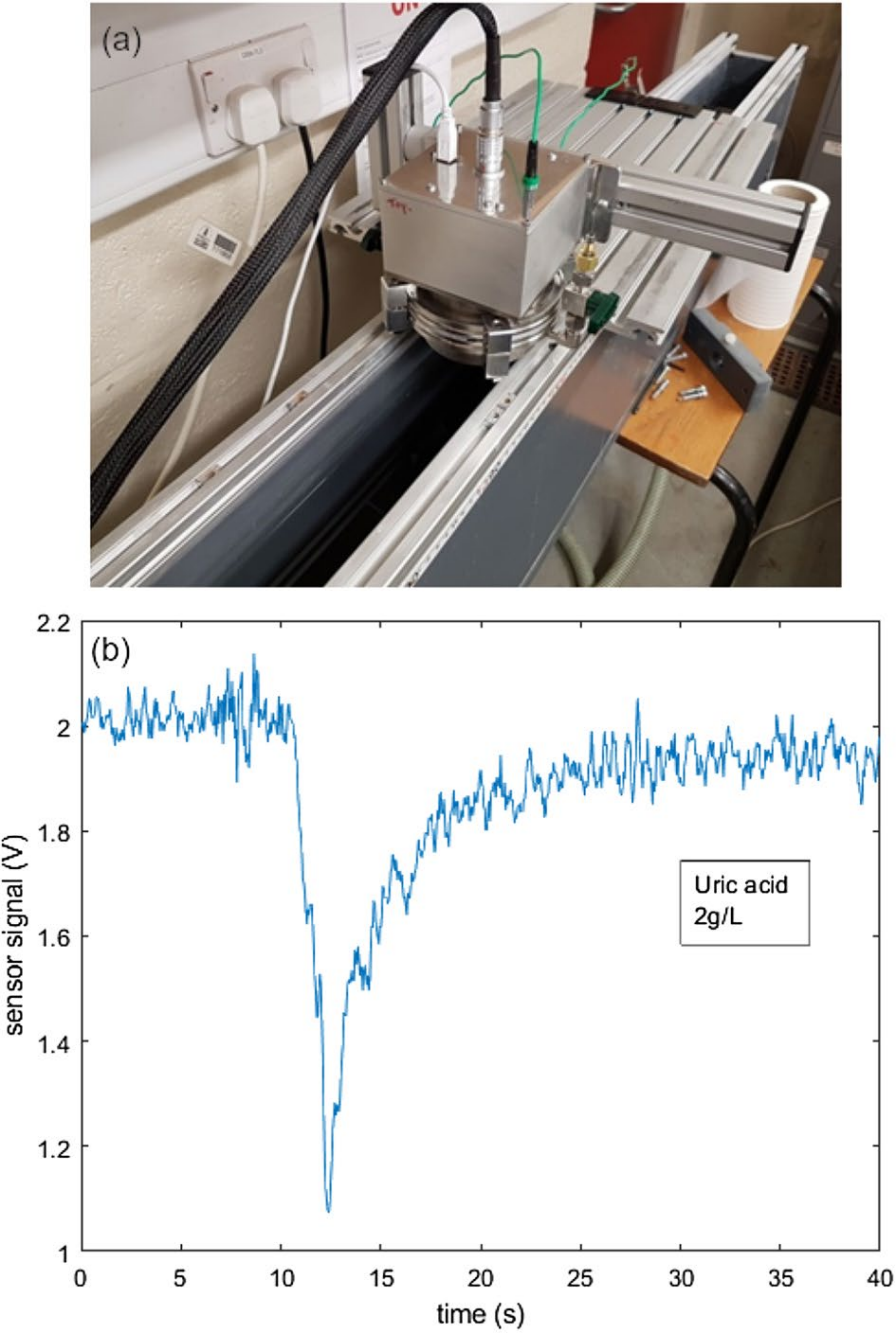
Photograph (**a**) of the sensor setup in a flume. The plot (**b**) shows a time series of sensor output. The pronounced dip at 10–12 s is due to arrival of uric acid solution deposited into the flume.

The sensor was also mounted above a 3 m long, 10 cm wide flume with a waterproofed LED light source immersed in the channel (Fig. 5). The LED had a power output 1 mW at 250 nm. A pump created a constant water flow in a closed loop. The flow rate was roughly 1 m/s at the surface, while at the bottom of the channel the flow was slightly slower with more turbulence. Unpurified water (direct from the main building supply) was used. The vertical optical path length through the water from LED to sensor was approximately 10 cm. With no contaminants added, there is still some absorption by the unpurified water. Fluctuations in the sensor signal were observed due to ripples and turbulence which may also involve refraction effects. 40 ml batches of contaminant solutions (tryptophan, uric acid and peptone) were added at the top of the flume. Typically a reduction in signal was observed after a few seconds as the contaminant passes the sensor, with the signal not returning to its pre-contaminated level. An example time series is shown in Fig. 5b for contamination by uric acid solution (2 g/l). For some samples a second discrete signal dip (less pronounced than the first) was observed as a more concentrated region of contaminant arrived at the sensor after a full loop through the flume. The “permanent” signal reduction, when the contaminant is evenly spread through the flume water, depended on the concentration and absorbance of the contaminant, as expected. Some further data are shown in the Supplementary Material.

## Discussion

The behaviour described above can be quantified with a simple Beer–Lambert absorption model using the data shown in Fig. 2. We write the wavelength-dependent transmission as a product of the material transmittance and the combined spectral response of the apparatus. Setting the emission spectrum as $I(\lambda )$, detector responsivity $Q(\lambda )$ and the apparatus transmittance as $T(\lambda )$, we obtain for the detected signal *S*,

1 $$\begin{aligned} S = \int _{0}^{\infty } I(\lambda ) Q(\lambda ) T(\lambda ) 10^{-A(\lambda )} \; \text {d} \lambda . \end{aligned}$$

Here, $A(\lambda ) = A_0(\lambda ) c / c_0$ where $A_0 = \epsilon (\lambda ) l c_0$ is the measured absorbance spectrum. The path length *l* = 10 mm is fixed, $c_0$ = 10 mg/L, *c* is the concentration and $\epsilon (\lambda )$ is the molar absorptivity. Note that there are no free parameters in the model, although we believe that the detector responsivity curve can vary due to the surface condition of the photocathodes. There may be other systematic errors in spectral parameters, which were not measured directly. The overall detector gain could vary slightly between contaminant measurements and normalisation measurements, which would multiply the whole transmittance curve by a fixed factor close to 1.

Results of this model are shown as solid lines in Fig. 3 and are in reasonable agreement with the data. Note that if the QE increases more rapidly than the assumed linear dependence below 235 nm, then the decrease of deuterium lamp transmittance would be stronger at higher KHP concentrations. However, for most of the substances of interest (Fig. 1) the short wavelength response (below 220 nm) is not as important as differences at longer wavelengths. Also, the deuterium lamp response is rather broadband (around 50 nm FWHM) compared to the LEDs ($\le 15$ nm FWHM). For these reasons, we turn to measurements comparing pairs of LEDs. Because the 310 nm LED lies close to the detector threshold its total response is weaker (Fig. 2), although some absorption signals could be measured at this wavelength. The 250 nm and 280 nm LEDs are most promising.

For a mixture of absorbing compounds with concentrations $c_i$ and absorbance spectra $A_{i}(\lambda )$, the total absorbance becomes

2 $$\begin{aligned} A(\lambda ) = \sum _{i} c_i \, A_{i}(\lambda ) / c_0. \end{aligned}$$

This model was used to produce the solid lines shown in Fig. 4 which agree reasonably well with the experimental data. The model suggests a slightly higher transmittance at 280 nm than 250 nm for the KHP–clothianidin mixture whereas the experimental data are nearly coincident at the two wavelengths. The opposite trends for 250 nm and 280 nm are nicely reproduced for the tryptophan–thiamethoxam mixtures, although the increase in transmittance is sharper in the experiment than in the model. The error bars in the experimental transmittance are quite small, around the size of the symbols, and so the discrepancies between model and data in Figs. 3 and 4 are likely due to systematic inaccuracies in the assumed spectral data, especially the QE curve shape, which may vary between photocathodes or even evolve with time during UV exposure. Note that we do not distinguish between absorption, fluorescence and scattering in the present work, presenting only normalised transmission intensity in the concentration-dependent data. Fluorescence may be important for some contaminants such as tryptophan and could readily be included in a more sophisticated model. Nonetheless, these experiments provide a clear demonstration of how the natural response curve of the threshold detector combined with a simple two-colour measurement can provide discrimination between two contaminants in water.

For an unknown mixture of two contaminants with ratio *r* (e.g. horizontal axes of Fig. 4) and total concentration *c*, one can write the signal at two wavelengths as $S_1 = f(c) g_1(r)$ and $S_2 = f(c) g_2(r)$ where *f*(*c*), $g_1(r)$ and $g_2(r)$ can be found experimentally or from a model. The measured ratio $S_1 / S_2 = g_1(r) / g_2(r)$ then gives *r*, thence *c* via $f(c) = S_i / g_i$. More complex mixtures would require more wavelengths, and the procedure would be most effective where the target compounds have distinct absorption features in different parts of the wavelength range, as in Fig. 4b. Practical limits to the number of wavelength channels would arise from the inherent spectral width of LED sources, the minimum wavelength available (around 245 nm at present, with a significant increase in cost at shorter wavelengths) and the wavelength window accessible within the water transparency window defined by the detector threshold. The measurements can readily be extended to lower contaminant concentrations by increasing the optical path length or measurement time, or by improving the signal-to-noise performance of the sensor. As with all optical measurements, however, turbidity could be problematic. We have also tested the absorption of natural water in the same experimental system, using local river and lake samples (see supplementary information). For turbid water, strong broadband absorption reduces the transmittance below the levels due to contamination, as expected, which would make measurements more difficult. Broadband absorption due to organic matter in the less turbid natural water sample is significantly smaller, allowing more reliable contaminant measurement at specific wavelengths. Even in the presence of natural organic absorbers, the UV absorption measurement could be employed as a dynamic measure of changes rather than as an absolute concentration measurement.

## Conclusions

Absorbance curves for a range of contaminants in water have been measured. Clothianidin, thiamethoxam, uric acid and tryptophan have distinct absorption features in the 250–300 nm range, where water transparency is high and UV LEDs can be used. KHP shows increasing absorption below about 260 nm but metaldehyde is quite weakly absorbing even down to 200 nm. Measurements of total transmittance have been made using a thresholding detector, UV LEDs and a deuterium lamp. These suggest that some useful spectral sensitivity can be obtained without the need for either filters or dispersing optics. In particular, through judicious choice of LED wavelengths the concentrations of different contaminants could be inferred from an absorption measurement; data were shown for tryptophan–thiamethoxam mixtures. Because of the simplicity of the setup, measurements can be made in real time. A demonstration of the contaminant response in a 3 m recirculating flume was given, showing clear changes of absorption over a timescale of seconds as regions of more contaminant pass by the detection point before spreading out to an even concentration. This type of measurement could be performed at all wavelengths across the water transparency window, with LED sources presently covering 250 nm and upwards, and could find application in a range of water quality situations, from catchment water monitoring to real-time analysis of produced water.

## Methods

Solutions of various water contaminants were made at different concentrations in laboratory grade deionised water. Some natural water data were also obtained (see Supplementary Material) and the flume data were obtained with unpurified water from the building’s supply. The standard for UV spectrometry was 10 mg/L with several other dilutions measured to check concentrations. A Jasco V-660 UV–Vis spectrometer was employed with the solutions placed in standard quartz glass cuvettes of 10 mm optical path length. For the threshold absorbance measurements, UV LEDs supplied by ThorLabs Inc. were used. Solutions were placed in the same quartz glass cuvettes and mounted in front of a threshold UV detector. The transmitted intensity was measured for a series of dilutions of each contaminant, and several mixtures were also studied. The UV detector is based on a gaseous electron multiplier (GEM) operating in atmospheric pressure of argon. Electrons photoemitted from the GEM front face, around 10 mm by 10 mm in area and at ground potential, are accelerated through holes in the GEM on to an anode at around 1 kV positive potential. Multiplication of the photoelectron current takes place as electrons are accelerated through the argon gas and cause a collision cascade, providing a gain of around 10$^3$. A picoammeter mounted directly behind the GEM housing measures the total drain current which is proportional to the intensity of UV light arriving at the GEM front face, weighted by the quantum efficiency as a function of wavelength. Since photons below a wavelength threshold defined by the work function cannot generate photoelectrons the device acts as a high-pass energy filter (low-pass wavelength filter) and can be described as a visible-blind thresholding detector. The quantum efficiency increases as the wavelength decreases below the threshold. No change of detector response is observed with changes in ambient lighting and even under direct illumination by a high power white LED torch (5 W), no signal is measured.

## Supplementary Information

Supplementary Information
