# Supplementary material for: Ultraviolet absorption of contaminants in water
Source: Sci Rep. 2021 Feb 11;11:3682. doi: 10.1038/s41598-021-83322-w (PMC7878760; doi:10.1038/s41598-021-83322-w)
Supplement: Supplementary file 1 — Supplementary Information [file 41598_2021_83322_MOESM1_ESM.pdf]

Supplementary Material for **Ultraviolet absorption of contaminants in water**

Martin Spangenberg, James I. Bryant, Sam J. Gibson, Philip J. Mousley, Yorck Ramachers, and Gavin R. Bell (gavin.bell@warwick.ac.uk)

1. Natural water

Samples of water were obtained from two lakes on the University of Warwick campus, from a stream on campus, and from the River Sowe in Coventry (with fast flow after heavy rain). Lake sample 2 had high turbidity (visible sediment) while other samples were visibly tinted compared to lab or tap water. UV transmittance in the standard geometry was measured as shown in the table.

	Deuterium Lamp	250 nm LED	280 nm LED
Lake 1	0.66	0.64	-
Lake 2	0.19	0.13	-
Stream	-	0.82	0.85
River Sowe	-	0.68	0.67

The transmittances of a given sample are similar at different wavelengths, consistent with broadband absorption due to sediment and organic materials in natural water. The tabulated values can be compared with the transmittances measured in Figures 3 and 4 of the main paper. Clearly, the high turbidity of lake sample 2 would hinder distinguishing the absorption due to contaminants in all but the highest concentrations shown in the main paper. For the other samples with lower turbidity the transmittance is sufficiently high to allow such measurements, particularly measuring dynamic changes with time rather than absolute concentrations.

2. Additional flume data

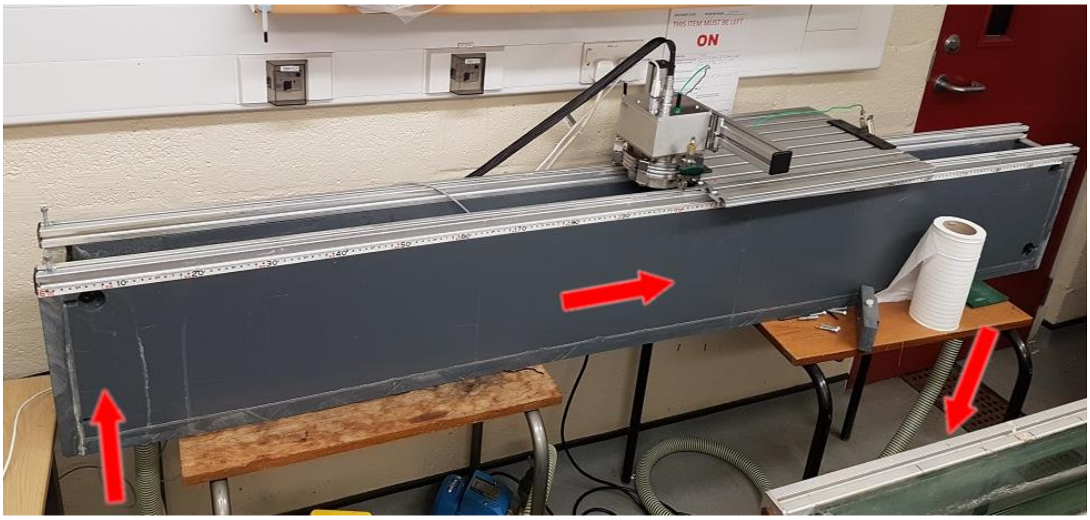

Figure S1: flume set-up. The sensor is seen mounted above the flume, with a waterproof LED light module immersed in the water beneath the sensor. The red arrows show the water flow cycle – water is returned to the left end of the flume via a pump. Contaminant was added to the left end of the flume by manually tipping in 40 ml of prepared solution.

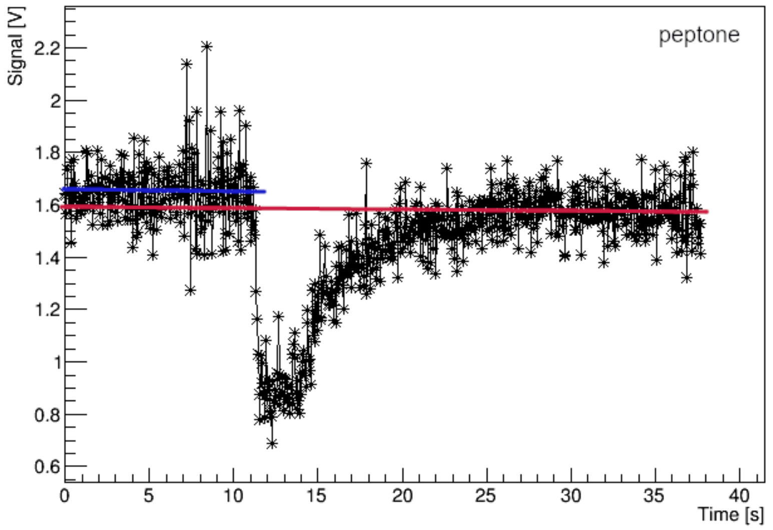

Figure S2: flume transmittance data for peptone (250 nm LED). Blue and red lines highlight the difference in steady transmittance after the first wave of contaminant has flowed past the sensor position at around 11 – 15 seconds and become well mixed with the flowing water.

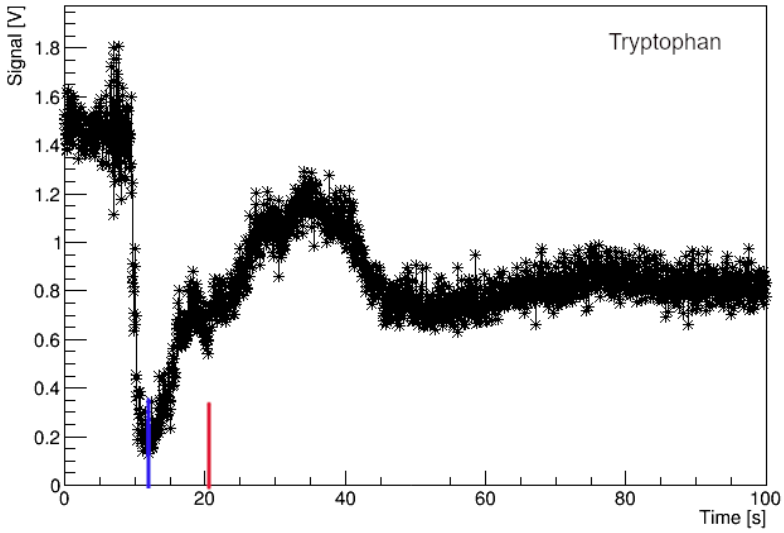

Figure S3: flume transmittance data for tryptophan (250 nm LED). Blue and red lines highlight the first and second arrivals of the contaminant at the sensor position, although the second is obscured by the combination of noise and increasing signal as the tryptophan become diluted throughout the volume of the flume. The origin of the drop back towards a steady state at around 40 seconds is not clear but may be related to turbulence and / or surface ripples.
